# Multiple Factors Related to the Secretion of Glucagon-Like Peptide-1

**DOI:** 10.1155/2015/651757

**Published:** 2015-08-20

**Authors:** XingChun Wang, Huan Liu, Jiaqi Chen, Yan Li, Shen Qu

**Affiliations:** ^1^Department of Endocrinology and Metabolism, Shanghai 10th People's Hospital, Tongji University, Shanghai 200072, China; ^2^Department of Urology, Zhenjiang First People's Hospital, Zhenjiang, Jiangsu 212002, China; ^3^Nanjing Medical University, Nanjing, Jiangsu 210029, China

## Abstract

The glucagon-like peptide-1 is secreted by intestinal L cells in response to nutrient ingestion. It regulates the secretion and sensitivity of insulin while suppressing glucagon secretion and decreasing postprandial glucose levels. It also improves beta-cell proliferation and prevents beta-cell apoptosis induced by cytotoxic agents. Additionally, glucagon-like peptide-1 delays gastric emptying and suppresses appetite. The impaired secretion of glucagon-like peptide-1 has negative influence on diabetes, hyperlipidemia, and insulin resistance related diseases. Thus, glucagon-like peptide-1-based therapies (glucagon-like peptide-1 receptor agonists and dipeptidyl peptidase-4 inhibitors) are now well accepted in the management of type 2 diabetes. The levels of glucagon-like peptide-1 are influenced by multiple factors including a variety of nutrients. The component of a meal acts as potent stimulants of glucagon-like peptide-1 secretion. The levels of its secretion change with the intake of different nutrients. Some drugs also have influence on GLP-1 secretion. Bariatric surgery may improve metabolism through the action on GLP-1 levels. In recent years, there has been a great interest in developing effective methods to regulate glucagon-like peptide-1 secretion. This review summarizes the literature on glucagon-like peptide-1 and related factors affecting its levels.

## 1. Introduction

**Figure 1:**
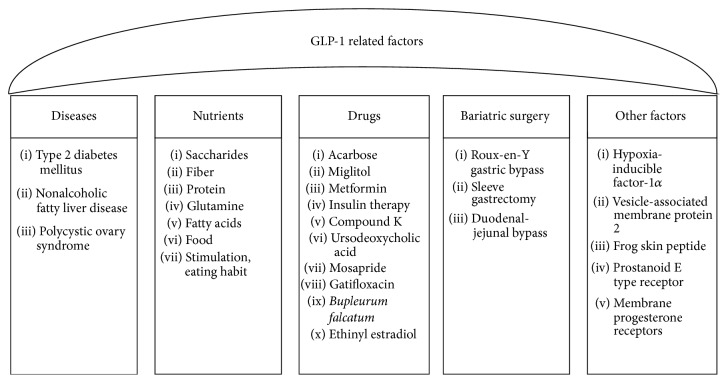
Levels of glucagon-like peptide-1 related factors.

Glucagon-like peptide-1 (GLP-1) is intestinal endocrine L cell-derived peptide. The receptors of GLP-1 are found in islet beta-cells, brain, cardiovascular system, and lung [1]. GLP-1 decreases blood glucose levels during hyperglycemia by stimulating insulin secretion and reducing glucose-dependent glucagon secretion [2–4]. GLP-1 promotes satiety and delays gastric emptying through central mechanisms, thereby reducing postprandial glucose levels [4]. The existence of a diurnal rhythm in GLP-1 secretion in response to an oral glucose load has been demonstrated in rats [5]. Study also showed a disruption of diurnal GLP-1 levels in overweight/obese subjects [6]. Two biologically active forms of GLP-1 exist: GLP-1 (7–37) and GLP-1 (7–36) amide. Biological activity of GLP-1 decreased soon after secretion due to decomposition by dipeptidyl peptidase-4 (DPP-4) [4]. Therefore, GLP-1 receptor agonists and DPP-4 inhibitors have been developed as novel types of antihyperglycemic drugs. Gastrointestinal taste receptors also regulate GLP-1 secretion [7–9]. Paracrine, nerve, and factors of neurohormone can also regulate the secretion of GLP-1 [10–12]. Plasma levels of GLP-1 were increased rapidly after nutrient ingestion, suggesting the existence of a proximal gut signal regulating GLP-1 release from the L cells of the distal small intestine [11]. The GLP-1 secretion is regulated by a complex neuroendocrine loop (proximal-distal endocrine loop), involving the enteric nervous system, the afferent and efferent vagus nerves, and the duodenal hormone glucose-dependent insulinotropic peptide (GIP) [11]. Overall, there are many factors affecting GLP-1 levels, including diverse types of nutrients, surgical procedures, drugs, and eating habits. This paper reviews factors affecting the levels of GLP-1 and they were showed in Figure 1.

## 2. Diseases Affecting GLP-1 Levels

Low GLP-1 level was an important risk factor for type 2 diabetes mellitus (T2DM) [13]. Fasting and postprandial GLP-1 levels were significantly lower in patients with T2DM than those with normal glucose tolerance (*P* = 0.02) [13]. The decreased levels of GLP-1 in obesity and T2DM are likely due to the reduction of GLP-1 secretion [14, 15]. Additionally, Vollmer et al. [16] suggested that GLP-1 secretion was not impaired in diabetic patients with well controlled blood glucose, while it was diminished in those with poor glycemic control or those with a longer duration of T2DM. The glycated serum (GS) and high levels of glucose (HG) may directly alter the function of neuroendocrine cells secreting this hormone by regulating different pathways of GLP-1 secretion [17]. Overall, it can be summarized that the levels of fasting GLP-1 and postprandial GLP-1 were reduced in subjects with T2DM compared to subjects with normal glucose tolerance [18–20]. However, there was also a study reporting that GLP-1 secretion in response to nutrient in T2DM patients was not affected [21]. Additionally, studies have found that glucose-induced GLP-1 secretion was remarkably decreased in NAFLD patients compared to healthy controls [22]. Polycystic ovary syndrome (PCOS) is related to insulin resistance, and the pathophysiologic mechanisms of PCOS are similar to those of T2DM [23, 24]. Therefore, patients with PCOS may have alterations in the incretin hormone response. Study showed that GLP-1 levels both at fasting and in response to a meal were significantly blunted in women with PCOS compared to healthy women (*P* = 0.022 and *P* = 0.028, resp.) and AUC for GLP-1 was also lower in PCOS (*P* = 0.012) [25]. GLP-1 concentrations have no significant difference in PCOS and control healthy women (CT) in the early phase of OGTT and then reached significantly lower levels in PCOS than in CT at 180 min (*P* < 0.05) which also exhibited a significantly different time-dependent pattern in PCOS (*P* < 0.002 for PCOS versus time interaction) [26]. These findings provide novel methods to augment GLP-1 levels for the treatment of obesity, T2DM, NAFLD, and PCOS, whereas this issue still causes dispute.

## 3. GLP-1 Levels and Nutrients

The levels of bioactive GLP-1 in fasting plasma usually range from 5 to 10 pmmol/L and increase approximately two- to threefold after meal [14]. Additionally, the postprandial peak of GLP-1 levels appears 20–30 minutes after a meal according to size and nutritional composition of it [14]. The mechanism leading to GLP-1 secretion may include the direct and indirect pathways. GLP-1 is secreted by the direct actions of luminal contents on the L cells in distal jejunum and proximal ileum [27]. Additionally, other mechanisms via “neural” or “upper gut” signals playing a second fiddle may stimulate GLP-1 secretion even before the luminal contents have reached into the proximities of L cells [27]. The direct exposure of L cells to luminal content appears to be the primary route for GLP-1 stimulation. Therefore, GLP-1 secretion is dependent on the nutrient composition of the meal and digestion of macronutrients [28–30]. A previous study has shown that the nutrients which may directly affect the secretion of GLP-1 after meals include glucose, triacylglycerol, fructose, and some proteins [31]. The intensity and degree of stimulation vary for different nutrients. The secretion of GLP-1 stimulated by lipids is the highest which is followed by glucose and amino acids. Since the macronutrient composition of a meal affects the secretion of GLP-1, it may provide novel nutritional alternatives of a meal for better management and prevention of diabetes, obesity, NAFLD, and some other metabolic related diseases in addition to the conventionally recommended diets [32–34].

### 3.1. GLP-1 Levels and Saccharides

Glucose can stimulate the secretion of GLP-1 in mice in energy-dependent manner [35]. GLP-1 secretion (total area under the curve (tAUC) per hour) was increased following oral glucose in human [36]. A randomized crossover study reported a 57% increase in plasma GLP-1 concentrations due to 50 g galactose and 2.5 g guar gum in combination with a standard breakfast in normal-weight women individuals [37]. Chitosan is widely applied in medical nutrition therapy as a dietary supplement and may be helpful in improving diabetes. Low molecular weight chitosan (LMWC) significantly increased GLP-1 secretion in human intestinal endocrine cells (NCI-H716) in a dose-dependent manner through a p38/MAPK-dependent signaling pathway [38]. Fructose intake increased plasma GLP-1 with a lower degree than isocaloric glucose in healthy humans [39]. Fructose stimulated GLP-1 secretion in a dose-dependent fashion by inducing ATP-sensitive potassium channel closure and subsequent cell depolarization in GLUTag cells [39]. Additionally, GLP-1 secretion was enhanced in rats receiving *α*-glucosyl-isoquercitrin (Q3G) plus fructooligosaccharides (FOS) compared with those receiving Q3G or FOS alone [40]. Q3G plus FOS also enhanced and prolonged high plasma GLP-1 level via direct stimulation of GLP-1 producing L cell indicating that a diet rich in Q3G and FOS may aid in the management of T2DM [41]. However, Sakamoto et al. [42] investigated the effects of a moderate sucrose diet (SUC) on incretin secretion in mice and their results showed that GLP-1 secretion 15 min after oral glucose administration was significantly lower in SUC-fed (38.5% sucrose) mice than in high-starch- (ST-) diet-fed or control mice [42]. This result indicated that consumption of a moderate sucrose diet may impair GLP-1 secretion.

### 3.2. GLP-1 Levels and Fiber

Nutrients and other intestinal hormones act as potent stimulants of GLP-1 secretion [43–45]. Dietary fiber is nondigestible carbohydrate which is categorized into three main subtypes: soluble (prebiotic, viscose), fiber, and insoluble fiber. The soluble (prebiotic, viscose) fiber is easily fermented into biomass, short-chain fatty acids (SCFA) chiefly acetic, propionic and n-butyric, lactate, and gases by the microflora of the large intestine. SCFA contact the intestinal flora and induce systemic effects. Although they serve as source of nutrients, SCFA also cause anorexia and induce GLP-1 release from L cells by acting on the G-protein-coupled free fatty acid receptor 2 in vitro [46]. One study examined the effects of probiotic supplementation on plasma gut peptide concentrations in healthy subjects and found that probiotics increased plasma GLP-1 concentrations while postprandial plasma glucose levels were decreased after the standardized meal [47]. Therefore, probiotics may be used as a useful tool in diabetic nutritional therapy because of the beneficial effects on appetite sensation and glucose excursion. Reports have also showed that resistant starch (RS) may increase plasma total GLP-1 concentrations in rodents although the exact mechanism is not fully understood [32, 48]. Keenan et al. [48] assessed the effects of nonfermentable and fermentable fibers on GLP-1 expression. Their results showed that only fermentable high amylose-resistant cornstarch- (RS-) fed rats had increased plasma GLP-1 levels. Thus, a bioactive functional food such as RS may be a natural approach for the treatment of obesity as it may affect overall energy balance through its ability to stimulate GLP-1 expression. Dietary fiber (DF) is an essential constituent of a healthy diet with high satiety. A recent study investigated the effect of DF on hormonal responses and found that solid meals enriched with soluble fiber, psyllium, strongly modified postprandial GLP-1 level in healthy subjects [49]. High cereal fiber intake (wheat fiber) also increased GLP-1 secretion. In hyperinsulinaemic patients, plasma GLP-1 concentration was 1.3 pmol/L higher than at baseline (about a 25% increase) and 1.4 pmol/L higher after 12 months compared with control [50]. Continuous ingestion of resistant maltodextrin (RMD) (a water-soluble nondigestible saccharide) increased GLP-1 secretion in normal rats is stimulated by its direct and indirect (enhanced gut fermentation) effects on GLP-1-producing cells [51]. Overall, probiotics and high-fiber diets have potential beneficial effects on gut hormones which can be used in the medical nutrition therapy of obesity and diabetes.

### 3.3. GLP-1 Levels and Protein

Dietary intake of protein may be an effective therapy to improve the glycemic response due to its ability to increase GLP-1 secretion [52–54]. The pathways involve peptide transporter-1 (PEPT1) and calcium-sensing receptor (CaSR) which is highly expressed in L cells. Oligopeptides stimulate GLP-1 secretion through PEPT1-dependent electrogenic uptake and activation of CaSR in mice [55]. Indeed, the proportion of protein in the diet has been shown to have an effect on GLP-1 secretion. Lejeune et al. [56] investigated levels of related hormones during a high-protein (HP) diet in 12 healthy women and found that GLP-1 concentrations were higher during consumption of the HP diet (30%, 40%, and 30% of energy from protein, carbohydrate, and fat, resp.) than during the adequate-protein diet (AP: 10%, 60%, and 30% of energy from protein, carbohydrate, and fat, resp.) after dinner. Different kinds of proteins and amino acids are not all similarly effective in stimulating GLP-1 release. Chen and Reimer [57] tested whether branched-chain amino acids (BCAAs) and dairy proteins had the same efficiency in regulating satiety hormone secretion in a human intestinal cell line (NCI-H716). Their results suggested that leucine, isoleucine, skim milk, and casein stimulated GLP-1 release at different levels. Skim milk, casein (not whey), 2% leucine, and isoleucine stimulated GLP-1 secretion by 1.6-fold, 2.5-fold, 4.7-fold, and 2.6-fold, respectively [57]. Dairy based products may therefore induce GLP-1 secretion due to the high proportion of BCAAs (21%) found in dairy. Another study in pigs also showed that adding dairy protein, a-casein or b-casein, to waxy maize starch increased GLP-1 release [58]. Meat hydrolysate (MH) and essential amino acids (EAAs) are powerful activators of GLP-1 secretion via activating extracellular signal-regulated kinase (ERK1/2) and p38 in the NCI-H716 cell line [59]. However, 14-day L-carnitine L-tartrate oral supplementation (LC) (3 g LC/day) has no effect on the total GLP-1 levels in response to oral glucose tolerance test (OGTT) in lean and overweight/obese males [60]. L-Arginine is an insulin secretagogue which increases GLP-1 levels and improves glucose clearance with this effect being ablated in Glp1r knockout mice indicating that L-arginine requires GLP-1 signaling in order to improve insulin signaling [61]. Thus, L-arginine-based nutritional or pharmaceutical therapies that improve glucose tolerance by increasing postprandial GLP-1 secretion may be used in diabetes and obesity. Collectively, nutritional strategies such as increased protein and other nutritional supplements such as L-arginine may be used to enhance postprandial GLP-1 secretion and may provide an alternative therapeutic approach in obesity and diabetes.

### 3.4. GLP-1 Levels and Glutamine

L cells are sensitive to changes in the glutamine content of meals and glutamine-based nutritional therapy may enhance GLP-1 secretion in diabetic and obese individuals. Reimann et al. [62] found that glutamine can act as a more potent GLP-1 secretagogue than glucose or other amino acids, increasing GLP-1 release 7.1 ± 0.7-fold at 10 mmol/L in GLUTag cells. Additionally, circulating GLP-1 concentrations were increased in healthy normal-weight volunteers (LEAN), obese individuals with T2DM or impaired glucose tolerance (OB-DIAB), and obese nondiabetic control subjects (OB-CON) after glutamine intake (peak concentrations at 30 min: LEAN: 22.5 ± 3.4; OB-CON: 17.9 ± 1.1; OB-DIAB: 17.3 ± 3.4 pmol/L), which may represent a novel therapeutic approach to stimulating insulin secretion in obesity and T2DM [63]. A study showed that 30 g glutamine (Gln-30) augmented postprandial active GLP-1 responses compared with control (water) in 15 T2DM patients and suggested that glutamine may be a novel agent for stimulating GLP-1 concentration and limiting postprandial hyperglycemia in T2DM [64]. Overall, these data suggest that single amino acid supplementation such as glutamine might be used as a potential nutritional therapy for T2DM and obesity. This method also requires further research on the long-term effects.

### 3.5. GLP-1 Levels and Fatty Acids

Free fatty acids act as signaling molecules and natural ligands for GPR [65]. Monounsaturated fatty acids (MUFAs), polyunsaturated fatty acids (PUFAs), and saturated fatty acids may alter the production of GLP-1. Tanaka et al. [66] examined the effects of acute and long-term administration of the natural ligand alpha-linolenic acid (alpha-LA) on plasma GLP-1 levels in rats with alpha-LA being able to increase GLP-1 levels. Several kinds of G-protein- coupled receptors (GPCR) have been identified in L cells including GPR119 [67]. The long-chain fatty acid derivate oleoylethanolamide (OEA) (10 micromoles/L) increased GLP-1 secretion from intestinal L cells through activation of the GPR119 fatty acid derivate receptor. Furthermore OEA-induced GLP-1 secretion was significantly reduced in mGLUTag cells transfected with GPR119-specific small interfering RNA [67]. Administration of 2-oleoyl glycerol (2OG) to healthy human volunteers activated GPR119 and caused plasma GLP-1 (0–25 min) to increase significantly when compared to the controls receiving oleic acid or vehicle [68]. Thus, GPR119 expressed in pancreatic islets and intestinal L cells has emerged as a new target for the treatment of T2DM as it may promote the secretion of GLP-1. Single administration of a novel GPR119 agonist (HD0471953) showed increased GLP-1 that may be a potentially promising antihyperglycemic agent for the treatment of patients with T2DM [69]. Additionally, an omega-3 unsaturated fatty acid metabolite, 5-hydroxy-eicosapentaenoic acid (5-HEPE), was a potent agonist for GPR119 and enhanced glucose-dependent insulin and GLP-1 secretion in mouse and it may play a protective role against diabetes [70]. GPR120 is abundantly expressed in the pancreas and intestine [71]. GPR120-mediated GLP-1 secretion induced by dietary free fatty acids (FFAs) may be effective in the treatment of diabetes as GPR120 is abundantly expressed in the intestine acting as a receptor for unsaturated long-chain FFAs [72]. Gpr40 is expressed in endocrine cells and beta-cells and Gpr40 mediates FFA-stimulated insulin secretion from beta-cells not only directly but also indirectly through regulation of GLP-1 secretion [73]. GPR40 agonists may represent a novel therapeutic strategy for the treatment of T2DM. One study found that administration of a novel GPR40 agonist (AS2575959) can increase GLP-1 levels [74]. Ingestion of a virgin olive oil-based breakfast (monounsaturated fat, MUFA; Mediterranean diet) increased GLP-1 concentration as compared with an isocaloric carbohydrate- (CHO-) rich diet in insulin-resistant subjects [75]. Docosahexaenoic acid (DHA) also has potential as an antidiabetic agent. Shida et al. [76] found that the plasma GLP-1 concentration of diabetic KK-A(y) mice increased after long-term DHA administration and this had a significant hypoglycemic effect. Colon targeting of DHA may provide a strategy for improving impaired glucose tolerance in T2DM by augmenting GLP-1 release. However, intake of abundant saturated fatty acids induces endoplasmic reticulum (ER) stress in the mouse intestinal L cell line (GLUTag cells) and decreases GLP-1 secretion [77].

### 3.6. GLP-1 Levels and Food

There are some alternatives for intake of some foods containing other dietary ingredients which increase endogenous GLP-1 secretion from intestinal L cells. Huang et al. [78] provided evidence that wild bitter gourd (BG) stimulated GLP-1 secretion involving certain bitter taste receptors and/or PLC *β* 2-signaling pathway in vitro, which in part contributes to the antidiabetic activity of BG through an incretin effect. Additionally, study found that a yellow pigment isolated from the rhizomes of* Curcuma longa* L. (curcumin) increases GLP-1 secretion in GLUTag cells through the Ca(2+)-Ca(2+)/calmodulin-dependent kinase II pathway and was independent of extracellular signal-regulated kinase, PKC, and the cAMP/PKA-related pathway [79]. Oral administration of the ZeinH (dietary protein hydrolysate prepared from corn zein) (2 g/kg) significantly increased plasma GLP-1 level and reduced glycemic response under the oral glucose tolerance test in normal Sprague-Dawley male rats and diabetic Goto-Kakizaki (GK) male rats [80].

### 3.7. GLP-1 Levels and Stimulation: Eating Habit

Factors beyond nutrients could contribute to the regulation of GLP-1 secretion. The combination of electrical stimulation (E-stim) and nutrient infusion significantly increased plasma GLP-1 levels when compared to nutrient infusion alone in either the ileum or the duodenum. And mechanical stimulation (M-stim) plus nutrient infusion significantly increased GLP-1 over nutrient infusion or M-stim alone in the duodenum, but not the ileum [81]. Additionally, there was a relationship between mastication and GLP-1 secretion. Sonoki et al. [82] compared the levels of plasma active GLP-1 concentrations after young healthy volunteers ate a test meal either by usual eating (control), unilateral chewing, quick eating, or 30-time chewing per bite. The results showed that plasma active GLP-1 concentrations did not change by unilateral chewing or quick eating but did increase with increasing chewing per bite. They also tested chewing 30 times per bite on plasma active GLP-1 concentrations in 15 patients with T2DM but there was no significant difference compared with usual eating. Further studies are needed to explore the long-term effects of eating habits and other life style modifications on GLP-1 secretion and plasma levels.

## 4. GLP-1 Levels and Drugs

### 4.1. GLP-1 Levels and Hypoglycemic Drugs

Some kinds of oral hypoglycemic drugs except for DPP4-inhibitors and GLP-1 receptor agonists affect plasma GLP-1 levels. The benefits of the alpha-glucosidase inhibitor (AGI) acarbose on cardiovascular risk may be associated with its stimulation of GLP-1 secretion. A 24-week treatment with acarbose led to significantly increased levels of both fasting and postprandial GLP-1 as well as significantly increased nitric oxide (NO) levels and nitric oxide synthase (NOS) activity for those patients in whom postprandial GLP-1 levels were increased in 24 newly diagnosed patients with T2DM [83]. Another AGI, miglitol, has the ability to influence bile acids (BAs) metabolism and improve insulin resistance and obesity. Miglitol enhanced active GLP-1 secretion into the portal blood and there was a positive correlation between active GLP-1 levels in diabetic mice [84]. The mechanism of the effects of AGI on GLP-1 secretion is that it has the inhibitory effects on the proximal carbohydrate absorption, allowing the distal gut to be exposed to unabsorbed carbohydrates, where L cells are densely distributed potentiating GLP-1 secretion [85]. Animal experiment showed that miglitol also activates duodenal enterochromaffin (EC) cells, possibly via sodium-glucose cotransporter (SGLT) 3, and mediates GLP-1 secretion through the parasympathetic nervous system [85]. Metformin was also reported to increase plasma intact GLP-1 concentrations in T2DM subjects [86]. Stimulation of GLP-1 secretion and reduction of soluble dipeptidyl peptidase-4 activity make contributions to this [86]. A study evaluated T2DM subjects on and off metformin monotherapy with results suggesting that metformin withdrawal was related to a reduction of active and total GLP-1 [87]. PCOS treatment of 40 women with 8 months of metformin 1000 mg twice daily increases the levels of GLP-1 [88]. Additionally, insulin therapy also has the ability to improve the GLP-1 concentrations in T2DM. One study found that in 26 patients with T2DM a short-term intensive insulin therapy for 10–14 days demonstrated significantly increased GLP-1 levels and AUC [89].

### 4.2. GLP-1 Levels and Other Drugs

Compound K (CK) has antidiabetic effects through as of yet incompletely understood mechanisms. CK has multiple biological functions via GLP-1 secretion and TGR5 activation. Kim et al. [90] found that treatment of NCI-H716 cells with 10, 50, and 100 *μ*M CK significantly increased GLP-1 secretion. However, transfection of NCI-H716 cells with TGR5-specific siRNA significantly inhibited CK-induced GLP-1 secretion indicating the importance of intact TGR5 signaling for the actions of CK. Bile acids play a well-known role in postprandial glucose response by stimulating GLP-1 secretion via the G-protein-coupled receptor. Ursodeoxycholic acid (UDCA) is a widely used therapeutic agent in liver diseases which may increase bile-induced GLP-1 secretion. Murakami et al. [91] investigated incretin and insulin secretion after a meal with or without UDCA in 7 nondiabetic Japanese subjects and found that UDCA intake resulted in higher GLP-1 secretion (AUC of 0–60 min after meal without UDCA, 450 ± 162 mmol·min/L; with UDCA, 649 ± 232 mmol·min/L, *P* = 0.046). Mosapride citrate is a selective agonist of the 5-hydroxytryptamine (5-HT)_4_ receptor, which is typically used to treat heartburn, nausea, and vomiting associated with chronic gastritis or to prepare for a barium enema X-ray examination and it may also have an antidiabetic effect by increasing GLP-1 secretion. Aoki et al. [92] examined the effect of the administration of mosapride citrate on plasma incretin levels in men with normal glucose tolerance (NGT) or impaired glucose tolerance (IGT) and showed that the AUCs of the plasma active and total GLP-1 levels were significantly higher in the M (mosapride citrate 20 mg) group than in the control (no drug) group. Ma et al. [93] evaluated GLP-1 responses to intraduodenal glucose in T2DM with results showing that the small intestinal glucose load is critical in determining GLP-1 responses. The GLP-1 responses to 120 min intraduodenal glucose infusions at 1 kcal/min (G1) and 2 kcal/min (G2) were minimal while responses to 4 kcal/min (G4) were much greater (*P* < 0.05 for each) [93]. Therefore, we deduced that the effect of mosapride on postprandial GLP-1 response may be secondary to its action on acceleration of gastric emptying. Additionally, Yu et al. [94] found that oral administration of gatifloxacin (100 mg/kg/day and 200 mg/kg/day) in rats for 3 and 12 days led to a marked increase in GLP-1 levels. Additionally, hexane fractions of* Bupleurum falcatum* (HFBF), which had been used as a medicinal herb, may activate the secretion of GLP-1 in NCI-H716 cells through the G *βγ* pathway [95]. GLP-1 levels both while fasting and in response to a meal are blunted in women with PCOS, which may contribute to the risk of impaired glucose tolerance and T2DM in polycystic ovary syndrome (PCOS) [74]. Hormone therapy may also have an effect on the GLP-1 levels. Women with PCOS who were lean and had normal glucose tolerance were treated with ethinyl estradiol 30 *μ*g/drospirenone 3 mg (EE/DRSP) for 3 months and had significantly reduced fasting and postprandial levels of GLP-1 and a decreased AUC for GLP-1 [25].

### 4.3. Other Factors Related to GLP-1 Levels

Obese adipose tissues are hypoxic and express hypoxia-inducible factor- (HIF-) 1*α*. However, deletion of HIF-1*α* in adipocytes may improve glucose tolerance by enhancing insulin secretion through the GLP-1 pathway. Kihira et al. [96] suggested that serum GLP-1 levels were increased in the adipocyte-specific HIF-1*α* knockout (ahKO), which demonstrated increased GLP-1 secretion from intestinal L cells. Vesicle-associated membrane protein 2 (VAMP2) also plays an important role in GLP-1 exocytosis from the GLUTag, and an improved understanding of the mechanisms governing GLP-1 secretion may lead to new approaches to enhance GLP-1 levels in T2DM [97]. Additionally, skin secretions of several frog species contain a component which can increase insulin secretion. Ojo et al. [98] suggested that frog skin peptide scans act as potential therapeutic agents for the treatment of T2DM by stimulating GLP-1 release and directly increase insulin secretion. There is abundant expression of prostanoid E type receptor (EP4) on mouse enteroendocrine GLUTag cells and administration of EP4 agonists to mice significantly increased plasma GLP-1 levels secreted from L cells [99]. Additionally, enteral progesterone administration can increase plasma levels of GLP-1 and intestine-restricted activation of membrane progesterone receptors may suggest a potential therapeutic approach for stimulation of incretin hormone secretion and control of glucose homeostasis [100]. Interestingly, an independent peripheral clock exists in the L cells which drives a circadian rhythm governing GLP-1 secretion in rats and thyrotroph embryonic factor and protein tyrosine phosphatase 4a1 altered GLP-1 secretion [5]. Thus, increasing GLP-1 levels by modifying these factors may represent novel approaches for the management and treatment of obesity and T2DM.

## 5. GLP-1 Levels and Bariatric Surgery

### 5.1. GLP-1 Levels and Roux-en-Y Gastric Bypass

GLP-1 has multiple effects on metabolism including increased insulin secretion and reduced food intake. Some bariatric surgery can serve to increase postprandial secretion of GLP-1 significantly which leads to the beneficial effects of reducing dietary intake, decreasing weight, and improving blood glucose. Roux-en-Y gastric bypass (RYGB) improves glycemic control in part through increased GLP-1 release. Severely obese glucose-tolerant individuals underwent RYGB with results indicating that GLP-1 secretion increased during postoperative OGTT at 3 months [101]. Mimicking the duodenal component of RYGB by implantation of a 10 cm endoluminal sleeve device (ELS-10) in diet-induced obese (DIO) rats also induced enhanced postprandial GLP-1 secretion and improved glucose tolerance and insulin sensitivity out of proportion to the effects of weight loss alone [102]. Additionally, plasma GLP-1 concentrations were increased with RYGB after 8 weeks in Sprague-Dawley rats [103]. After RYGB, food passes without hindrance into the small intestine and the rapid exposure of the gut epithelium leads to the enhanced GLP-1 secretion in RYGB patients compared to control subjects [104]. The anatomic explanation for RYGB augmenting secretion of GLP-1 is an observed 4.9-fold increase in GLP-1 cell density in the jejunum of eighteen nondiabetic patients with obesity 12 months after RYGB [105]. Exaggerated GLP-1 secretion is likely to be the main mechanism in the weight loss after RYGB. However, GLP-1 receptor (GLP-1R) signaling is not necessarily for weight loss after RYGB in rodents. Obese GLP-1R-deficient mice lost the same amount of body weight and fat mass compared with wild-type mice [106]. The effects of RYGB on energy and glucose metabolism still exist in two mouse models of functional GLP-1 deficiency which showed that GLP-1, acting through its classical GLP-1 receptor or its bioactive metabolites, does not seem to be associated with the effects of RYGB on weight loss and glucose homeostasis [107]. However, [107] demonstrated that RYGB utilizes taste receptor signaling via *α*-gustducin to increase peripheral GLP-1 secretion. It also demonstrated that RYGB-induced weight loss in the absence of enhanced GLP-1 secretion is enough to render the effects of RYGB on antidiabetic aspects. It is thought that the beneficial effects of RYGB are expressed through complex mechanisms that require comprehensive methods for identification.

### 5.2. GLP-1 Levels and Sleeve Gastrectomy

Sleeve gastrectomy is a relatively new operation that has shown benefits on T2DM and weight loss. Vertical sleeve gastrectomy (VSG) is a common type of bariatric surgery for weight loss in obesity and T2DM. Direct infusion of liquid nutrients into the duodenum has been shown to significantly increase GLP-1 release in VSG, indicating that increase in GLP-1 secretion after VSG is the result of both greater gastric emptying rates and altered responses at the level of the intestine [108]. Additionally, increased GLP-1 release has been suggested as a possible mechanism underlying the improvement in T2DM after laparoscopic sleeve gastrectomy (LSG). LSG led to increased GLP-1 secretion during OGTT and markedly increased intestinal motility at 15 and 30 min during OGTT at 3 months after the surgery in 12 obese patients with a body mass index >35 kg/m^2^ [109]. One study found that SG and BPD markedly enhanced GLP-1 responses levels in patients [110]. Pylorus-preserving pancreatoduodenectomy in 10 overweight patients without T2DM also resulted in a remarkable increase in GLP-1 levels in response to a mixed meal [111].

## 6. GLP-1 Levels and Duodenal-Jejunal Bypass

Expedited biliopancreatic juice flow to the distal gut was associated with increased GLP-1 secretion which may partly explain the metabolic benefits of duodenal-jejunal bypass (DJB) [112]. DJB-operated diabetic rats exhibited higher glucose-stimulated GLP-1 secretion than the duodenal-jejunal anastomosis (DJA) group postoperatively [112]. Overall, metabolic surgeries are effective in improving glucose metabolism and weight loss may in part be due to the enhanced GLP-1 levels.

## 7. Conclusion

Multiple factors are related to the secretion of GLP-1. Using various methods to increase the secretion of GLP-1 may provide alternative therapeutic options to treat metabolic disorders such as obesity, diabetes, and NAFLD which may be due to the lack of GLP-1. Modifying eating habits, food components, and some other factors to regulate GLP-1 levels may promote better management and treatment of these disorders. The mechanism and long-term effectiveness of factors affecting the regulation of GLP-1 are still not fully understood. Thus, further researches are still needed to assess the therapeutic potential of GLP-1 mediated therapies.
